# Effectiveness of Home-Based Stretching and Strengthening Training for Improving Flexibility, Strength, and Physical Function in Older Adults with Leg Tightness and/or Suspected Sarcopenia

**DOI:** 10.3390/sports13030065

**Published:** 2025-02-21

**Authors:** Pornpimol Muanjai, Sirawee Chaovalit, Nongnuch Luangpon, Wirasinee Srijunto, Pongrung Chancharoen, Juntip Namsawang, Piyapong Prasertsri, Sigitas Kamandulis, Tomas Venckunas, Orachorn Boonla

**Affiliations:** 1 Department of Physical Therapy, Allied Health Sciences Faculty, Burapha University, Chonburi 20131, Thailand; pornpimolm@buu.ac.th (P.M.); nongnuchl@go.buu.ac.th (N.L.); wirasinee@go.buu.ac.th (W.S.); juntip@go.buu.ac.th (J.N.); 2 Department of Physical Therapy, Faculty of Medicine, Prince of Songkla University, Songkhla 90110, Thailand; sirawee.c@psu.ac.th; 3 Faculty of Allied Health Sciences, Burapha University, Chonburi 20131, Thailand; pongrung@go.buu.ac.th (P.C.); piyapong@go.buu.ac.th (P.P.); 4 Institute of Sport Science and Innovations, Lithuanian Sports University, 44221 Kaunas, Lithuania; sigitas.kamandulis@lsu.lt (S.K.); tomas.venckunas@lsu.lt (T.V.)

**Keywords:** home-based exercise, stretching, eccentric exercise, Timed Up-and-Go, muscle thickness, stiffness

## Abstract

Background/Objectives: The aim of the present study was to assess the effectiveness of flexibility or strengthening exercises to improve flexibility, strength, muscle architecture, and functional performance in older adults with leg tightness and/or suspected sarcopenia. Methods: Ninety adults with leg tightness and/or suspected sarcopenia (age: 66.8 ± 4.9 years) were randomly allocated to two subtypes of intervention at home: resistance-band exercise (RE) or eccentric exercise (ECC) for those with weakness; static or dynamic stretching for those with tightness; and static stretching plus ECC or no exercise for those with both muscle tightness and weakness. The program consisted of 3–6 weekly sessions over eight weeks. Blinded outcome assessments before and after the eight-week program and at the three-month follow-up included mobility performance via Timed Up-and-Go (TUG), and flexibility and strength tests, as well as measurement of stiffness. Results: All groups had increased peak torque after eight weeks and improved TUG at the three-month follow-up (*p* < 0.05). Improved plantar flexor strength persisted at the three-month follow-up (*p* = 0.009). In addition, the RE and ECC groups had increased muscle thickness by 4.0 and 8.7% after eight weeks (*p* < 0.05). Hamstring flexibility increased in all exercise groups, except the RE group. Moreover, all six groups showed improved calf flexibility, whereas no changes in stiffness were noted. Conclusions: Increases in mobility performance, strength, and flexibility appeared due to learning effects and increased physical activity, rather than the specific training impact. However, strength-based programs may be recommended for older adults with suspected sarcopenia, as they provide additional benefits, such as short-lasting muscle hypertrophy.

## 1. Introduction

A decrease in physiological function is an inevitable aspect of aging. Currently, no definitive strategy has been identified to counteract its effects fully. Several factors contribute to functional decline in older adults, including mitochondrial dysfunction, telomere damage, epigenetic dysregulation, DNA damage, and lipid accumulation [1,2,3]. Changes in these molecular factors may be precipitated by a sedentary lifestyle, and may contribute to a loss of strength, aerobic endurance, and mobility [4]. Age-associated loss of muscle mass, which is known as sarcopenia, is one of the main contributing factors to muscle weakness. Sarcopenia is also a major consequence of chronic diseases, and results in disability and loss of independence [5]; moreover, it is associated with increased all-cause mortality [6]. In addition to muscle weakness, an age-related decline in flexibility can be attributed to several factors, including collagen cross-linking, increases in intramuscular adipose tissue and extracellular water content, and a reduction in collagen fibril diameter [7,8,9].

Exercise may mitigate this physical degeneration associated with aging, and sarcopenia can be effectively managed through resistance-based exercise. Home-based resistance exercises using elastic bands have been shown to improve lean mass, strength, and muscle quality in older adults [10]. Eccentric exercise, which involves muscle lengthening under load, is particularly advantageous for the elderly because of its lower metabolic cost and perceived effort, despite higher training loads. In fact, even low-intensity, no-equipment eccentric muscle contractions are more effective than concentric training in enhancing physical function and health [11]. Moreover, resistance exercises performed across a wide range of motion (ROM), including eccentric training, have been demonstrated to offer additional benefits in terms of flexibility [12,13,14].

In contrast to resistance exercises, which primarily enhance muscle strength, flexibility is predominantly improved through stretching interventions. Both acute and chronic static stretching have been shown to increase ROM in older adults [15,16]. These improvements may be partly attributed to reductions in musculotendinous unit (MTU) stiffness and muscle or nerve stiffness, and/or increased fascicle length [17,18,19]. Stretching, particularly when combined with rhythmic movements, may offer additional benefits, such as enhanced balance [20,21] and physical function [19,22]. For example, dynamic hamstring stretching involving seven repetitions resulted in a 45% improvement in hamstring flexibility, a 10% increase in knee strength torque, and a 14.3% reduction in the time to complete the Timed Up-and-Go test among older women [22].

Home-based exercise represents a practical and cost-effective approach to improve health, and provides a safe and feasible solution for older adults [23,24]. As noted, older adults may experience either singular or multiple impairments, and individualized supervised exercise training targeting specific issues could offer additional benefits beyond the primary objectives. Despite these advantages, there remains limited evidence regarding the effectiveness of home-based flexibility or strengthening exercises on outcomes such as muscle architecture, flexibility, MTU stiffness, strength, and functional performance in older adults with muscle weakness and/or tightness. The aim of this study was to address this gap by evaluating whether home-based flexibility exercises (static stretching) or strengthening exercises (eccentric training) produce greater improvements in these outcomes than dynamic stretching or elastic-band strengthening. Our hypothesis was that both types of home-based exercise would improve flexibility, muscle ultrasound measures, and strength. Additionally, we expected that individuals with multiple weaknesses would tend to have lower mobility, and would experience greater improvements in functional performance when participating in programs targeting both strength and flexibility. This knowledge allows for the implementation of more tailored and effective treatment strategies, thus optimizing their outcomes for older adults with varying degrees of muscle weakness or tightness.

## 2. Materials and Methods

### 2.1. Participants

**Figure 1:**
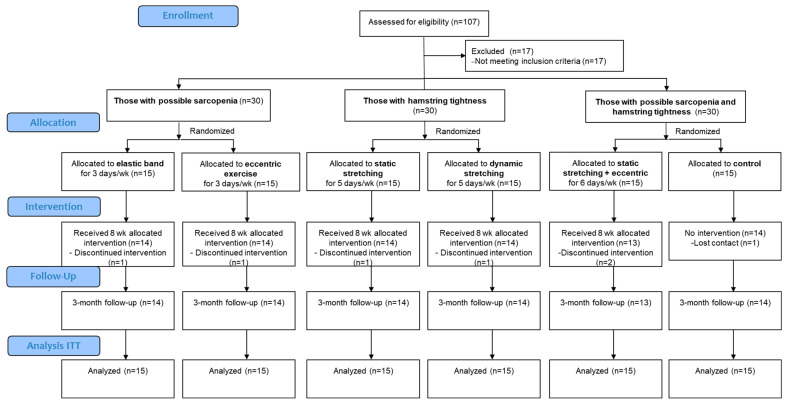
CONSORT diagram of study.

Older adults of both genders, aged between 60 and 75 years old, who were experiencing muscle tightness and/or weakness were recruited into this study (mean age, 66.8 ± 4.9 years; BMI, 23.1 ± 3.9 kg∙m^−2^). Figure 1 depicts the CONSORT flow diagram of the study, showing the formation of six subgroups, culminating in a total of 90 older adults with muscle tightness and/or weakness. Participants were recruited via advertainments from Chonburi Province, including the areas of Seansuk, Mueang, and surrounding communities. The inclusion criteria were as follows: individuals who were categorized as “low active” (engaging in less than 150 min of moderate physical activity level per week), had a BMI of less than 30 kg∙m^−2^, and passed the Physical Activity Readiness Questionnaire. Individuals with hamstring muscle tightness were identified as having a result of ≤80° on a passive straight-leg raise (SLR) test [25]. Those with generalized muscle weakness, which is potentially indicative of sarcopenia, were identified based on a calf circumference of less than 34 cm for males and less than 33 cm for females, or a Simple Questionnaire to Rapidly Diagnose Sarcopenia (SARC-F) score of ≥4. Moreover, a handgrip strength of below 28 kg for males and below 18 kg for females was used in this context, according to the guidelines of the Asian Working Group for Sarcopenia 2019 [26]. The exclusion criteria were as follows: uncontrolled hypertension or diabetes mellitus, decompensated heart failure, severe kidney disease, severe peripheral artery disease, neurological conditions that could interfere with exercise, abnormal electrocardiography findings, significant lower-body injuries within the past six months (e.g., fractures, dislocations, ruptures, and joint replacements), dependence on walking assistance, a history of lower-body pain exceeding 5/10 on the visual analog scale within the past six months, participation in stretching and resistance exercises in the preceding three months, treatments that could affect hemodynamic responses or muscle function, or missing more than 20% of the scheduled training sessions. All participants were provided with detailed information regarding the study protocol, and gave written informed consent before their participation. The study protocol received approval from the Research and Innovation Administration Division of the Burapha University Ethics Committee (IRB1-076/2566), in accordance with the principles outlined in the Declaration of Helsinki.

### 2.2. Study Design

Initially, the participants were familiarized with the testing apparatus at the Allied Health Science Faculty, Burapha University, 2–3 days before the baseline measurements were taken. The assessments were conducted in a specified order on the right leg, and included evaluations of muscle (body mass, biceps femoris [BF], vastus lateralis [VL], medial gastrocnemius [MG]) morphology, leg flexibility, musculotendinous unit (MTU) stiffness, leg muscle strength, and the Timed Up-and-Go (TUG) test. Measurements were collected at baseline, after an eight-week training protocol, and at the three-month follow-up. All assessments were performed by the same skilled and blinded investigators, to ensure consistency. The reliability of these measurements has been demonstrated in a previous study [19].

Participants in the groups with matched conditions (muscle tightness, muscle weakness, and both tightness and weakness) were randomly assigned to one of the subtypes of exercise intervention corresponding to each condition. Randomization was achieved using sequentially numbered, opaque, and sealed envelopes. The exercise interventions were as follows: home-based resistance-band exercise (RE) or eccentric exercise (ECC) for those with muscle weakness; home-based static stretching (SS) or dynamic stretching (DS) for those with muscle tightness; and a home-based combination of static stretching with eccentric exercise (SS + ECC) or an educated control condition (CON) for those with both muscle tightness and weakness.

During the first week of the exercise program, the participants met at the laboratory to perform exercises collectively, receive training on safety protocols, and learn how to monitor the signs of safety and progress while exercising at home. Each session commenced with a 7 min warm-up that involved self-paced walking. The exercise regimen consisted of 3–6 sessions per week over eight weeks, and targeted both legs. The home-based exercise protocols were detailed in a booklet using written and visual instructions, and the participants were provided with an exercise diary for program tracking. Research assistants engaged in real-time video calls at least once per week to supervise the home exercises, and the participants were required to attend laboratory sessions at least twice during the three-month follow-up period to discuss progress and their continuous engagement with the exercise sessions of the home program. After a three-month unsupervised period, all participants were asked to record the number of continuous sessions of exercise training in which they continued to engage. This study protocol was registered as a clinical trial (TCTR20240605001) “www.thaiclinicaltrials.org (accessed on 12 January 2025)”.

### 2.3. Interventions

#### 2.3.1. Home-Based Resistance-Band Training

The exercise program, which was designed based on the study reported by Nilsson et al. [10], consisted of the following three lower-body exercises: knee extension, knee flexion, and plantar flexion, which were all performed in a seated position. Each exercise was conducted with controlled movements, emphasizing a slow pace and a wide range of motion, while in a seated position. The participants performed 3 sets of 15 repetitions per exercise, with a 1 min resting interval between sets. Moreover, the participants were instructed individually regarding the use of the Rate of Perceived Exertion (RPE) scale, which is based on Borg’s original scale, and a metronome was provided to them to aid in maintaining the exercise tempo. The exercise intensity was progressively increased over the eight-week period; initially, the intensity was low (RPE: 6–11) in the first two weeks, subsequently advancing to a moderate intensity (RPE: 12–17) for 3rd–6th weeks, and ultimately to a high intensity (RPE: >18) in the last two weeks [11]. The bands were available in various resistances: yellow (1.32 kg), red (1.77 kg), green (2.27 kg), blue (3.22 kg), and black (4.40 kg). The tension was increased gradually throughout the study, to ensure continued adaptation. The participants engaged in the exercise program three times per week, on nonconsecutive days, for a total duration of eight weeks.

#### 2.3.2. Home-Based Eccentric Training

The training program was executed in three standing positions: one-leg squat, single-leg Romanian deadlift T-drop, and calf down with chair or wall support. This regimen was based on previous studies [11,27,28]. Specifically, the single-leg Romanian deadlift T-drop targeted the hamstrings [27], whereas the one-leg squat and calf down exercises addressed the knee extensors and ankle plantar flexors, respectively. To control the ROM during exercise, the single-leg Romanian deadlift T-drop began with the knee flexed at 10–15°, with the individual gradually leaning forward until reaching the maximum hip flexion ROM. The one-leg squat was performed slowly, with the knee bending to achieve 90° of flexion. The single calf down started from maximum plantar flexion and slowly descended to the endpoint of dorsiflexion. The exercises were performed using controlled movements, emphasizing slow and precise technique, for 3 sets of 15 repetitions each, with a 1 min resting interval between sets. The exercise intensity was progressively increased based on the RPE scale, as described previously. The program’s progression involved the optional use of a backpack for additional weight, to enhance intensity further [28]. The participants performed the training program three times per week on nonconsecutive days, over an eight-week period.

#### 2.3.3. Home-Based Static Stretching

After a standardized warm-up, the participants performed the stretching exercises as follows. For the knee extensors, the participants stood on one leg, using a wall or a chair for support. They were instructed to grasp the contralateral ankle while fully flexing the knee, and to increase hip extension if maximum tension of the knee extensors had not been achieved. For the knee flexors, the participants raised their leg with the knee kept straight and placed it on a chair or table. The stretch was maintained by making slight adjustments to the hip flexion angle, to ensure optimal stretching [29]. For the ankle plantar flexors, the participants began by standing on both legs, then stepped the leg to be stretched backward, while keeping the knee straight. The heel of the foot was allowed to contact the floor, with the contralateral knee slightly bent, to enhance the stretch. All stretching exercises were held at a constant maximum tension, ensuring that no pain was experienced while the muscles were relaxed as much as possible, for a duration of 30 s per stretch. Each stretch was performed 8 times, with a 15 s resting interval between repetitions. This stretching regimen was performed five times per week over a period of eight weeks.

#### 2.3.4. Home-Based Dynamic Stretching

After a standardized warm-up, the dynamic stretching exercises for the leg muscle groups were performed as follows. For the knee extensors, the participants performed the stretch in a standing position. They grasped the ankle of the involved leg while maintaining full knee flexion, then actively extended the hip to deepen the stretch. For the knee flexors, a closed kinetic chain hamstring stretch was employed, as described by Chen et al. [30]. The participants stood on the involved leg with the knee slightly flexed (10–15°), and leaned forward slowly until they reached the end of the hip flexion ROM. To maintain spinal alignment and balance, the contralateral leg was lifted into hip extension. The participant alternated between bending and extending the knee for 2 s each, thereby stretching the hamstring muscles. The intensity of the stretch was adjusted by modifying the forward-leaning angle of the trunk. For the calf muscles, the participants stood with one leg positioned at the edge of a stair, keeping the knee straight and allowing the heel of the foot to extend beyond the edge. They then alternated between performing ankle dorsiflexion while bending the contralateral knee. To enhance stability during the exercises, the participants used the back of a chair or a wall for support, especially during one-legged stances and forward-leaning positions. Each stretching exercise was performed until maximum tolerable tension was achieved without causing pain, and the exercises were repeated 15 times, for a total of 30 s, with continuous motion, using 8 sets, with a 15 s break between sets. The program was executed 5 times per week over eight weeks.

#### 2.3.5. Home-Based Static Stretching Plus Eccentric Exercise

After a standardized 7 min warm-up, the participants engaged in a structured exercise regimen consisting of static stretching and eccentric exercises. The program was designed to alternate between these two types of exercises, with the participants performing static stretching and eccentric exercises on separate days. Specifically, the participants completed static stretching for 3 days per week and eccentric exercises for 3 days per week, resulting in a total of 6 exercise sessions per week. This schedule was maintained for eight weeks.

#### 2.3.6. Control

The participants included in this group received information on general exercise recommendations for older adults, as outlined by the American College of Sports Medicine (ACSM) guidelines 2022. Moreover, the participants were asked to document their weekly physical activity levels in a booklet that was provided to them.

### 2.4. Outcome Measures

#### 2.4.1. Handgrip Strength

Handgrip strength assessment was used only for screening individuals with suspected sarcopenia, as it is commonly employed for this purpose. Handgrip strength assessment is commonly employed as a measure of maximum grip strength. In this study, a Baseline Hydraulic Hand Dynamometer (Fabrication Enterprises Inc., New York, NY, USA) was used to evaluate grip strength. Participants were seated with their arms extended in a neutral position, and were instructed to squeeze the dynamometer with maximal effort for a duration of 2–3 s. Three separate grip strength measurements were recorded for each participant, and the mean value of these measurements was calculated and used for subsequent analysis [10].

#### 2.4.2. Body Mass

Body mass was assessed using a BC-705N WH Tanita Body Composition Monitor (Tanita Corporation, Tokyo, Japan) at three time points: at the baseline, after an eight-week training protocol, and at the three-month follow-up.

#### 2.4.3. Timed Up-and-Go Test

The physical mobility performance was assessed by the TUG test [31], which measures the time that is required for a participant to stand up from an armchair, walk a distance of 3 m, navigate around a cone, and return to a sitting position on the chair. The setup of this test includes a firm chair with arms (seat height, 46 cm) and a cone placed 3 m away from the chair. The test procedure began with the participant seated in the chair with their back against the backrest, arms resting on their lap, and feet positioned just behind the distance marker on the floor. The participants received the following instructions: “On the word ‘go’, stand up, walk comfortably and safely to the cone on the floor, walk around the cone, return to the chair, and sit back down”. Timing started on the word “go” and stopped when the participant’s back rested against the chair upon returning. Each participant first completed a practice trial, followed by two recorded trials. The data from the two recorded trials were averaged for use in the data analysis [32].

#### 2.4.4. Muscle Architecture

B-mode ultrasonography was employed to capture muscle images of the right leg, using an M5 series ultrasound system (Shenzhen Mindray Bio-Medical Electronics, Shenzhen, China) with a linear 4 cm, 7.5 MHz probe (MSK preset). The ultrasound probe was positioned 15 cm above the popliteal line, with the participant in a relaxed prone position. For the BF muscle, images were captured in the longitudinal view, both at rest and at the endpoint of passive SLR procedures. This was carried out using a thin layer of ultrasound gel and minimal pressure applied by the probe. The thickness of the VL muscle was measured at the same thigh level in the supine position, using transverse imaging. The MG muscle was also imaged longitudinally, both at rest and at the endpoint of a passive ankle dorsiflexion test. For these measurements, the ultrasound probe was positioned 10 cm below the popliteal line. For each muscle, transverse views were also captured to assess echo intensity (EI), and each location was imaged twice. The ultrasound settings were kept consistent across participants, with a frequency of 10 MHz, the gain set at 54 dB, a dynamic range of 60 dB, and a depth of 5 cm. Muscle thickness (MT) was analyzed, with MT defined as the distance between the superficial and deep aponeuroses. Fascicle length (FL) was estimated by extrapolating the visible fascicles between the superficial and deep aponeuroses using Tracker 6.2.0 “https://opensourcephysics.github.io/tracker-website/ (accessed on 12 January 2025)” [33]. For each muscle, the averages of two images for MT and FL were calculated and used for further analysis.

#### 2.4.5. Flexibility

Leg flexibility measurements were performed as described in our previous study [19]. Hamstring flexibility was assessed using a passive SLR with a bubble inclinometer (Baseline, Fabrication Enterprises, Elmsford, NY, USA). The maximum angle recorded by the inclinometer was noted, and a longitudinal image of the BF muscle was captured using ultrasound. Calf flexibility was evaluated through passive ankle dorsiflexion (PDF) in a prone position, using a standard goniometer (Mahidol University, Bangkok, Thailand). Each test was performed three times, with a 15 s resting interval between attempts. The average of the two closest angles was used for further analysis.

#### 2.4.6. Passive MTU Joint Stiffness

A Biodex System 4 isokinetic dynamometer (Biodex Medical Systems, Inc., Shirley, NY, USA) was utilized for the passive assessment of MTU properties. The setup, testing protocol, and analytical procedures used for knee flexors and ankle plantar flexors were as delineated in our previous study [19]. In this study, a minor modification was made to the ROM testing for plantar flexor MTU stiffness: the ankle was positioned from 30° of plantar flexion to an endpoint of 30° dorsiflexion while seated. Passive stiffness was determined from the slope of the passive torque–angle relationship, corrected for the weight of the lower leg, by analyzing the increase in passive torque between 50 and 80% of the maximum ROM using a least-squares method [29,34].

#### 2.4.7. Leg Muscle Strength

Leg muscle strength was assessed in the right leg using a Biodex System 4 dynamometer (Biodex Medical Systems, Inc., Shirley, NY, USA), at a sampling rate of 100 Hz. Before the maximum isometric voluntary contraction (MIVC) procedure, the participants were secured in the dynamometer chair, and performed warm-up contractions at 50% of maximal effort for 2–3 repetitions, followed by a 2 min resting period. For the testing of knee extensors and flexors, the participants were positioned with the hip flexed to 85°, and the knee flexed to 70° for extensors and 50° for flexors. For ankle plantar flexion, the participants were seated with the hip flexed to 85°, the knee slightly bent at 10°, and the ankle in a neutral position (0°) [35]. During the MIVC, the participants were encouraged to “exert maximal force as hard as possible and hold for 3 s”, with each contraction repeated after a 1 min resting interval. The peak torque of each MIVC was recorded and analyzed for further evaluation.

#### 2.4.8. Level of Satisfaction with Exercise Program

The participants provided ratings of their satisfaction with the exercise program after eight weeks of training, using a Likert scale ranging from +3 to −3. On this scale, +3 denotes “extremely satisfied” and −3 indicates “very dissatisfied”.

### 2.5. Statistical Analysis

From the preliminary study, we determined that a sample size of 10 subjects per group would be sufficient to detect muscle strength differences with a statistical power of 80%, assuming a significance level of *p* < 0.05 and an effect size greater than 0.8. The sample size was subsequently increased by 50% (N = 15) in each group to account for expected dropout events. Similarly, Tarnopolsky et al. [36] suggested that a sample size of 10 participants per group is adequate to detect differences in body composition and strength.

Descriptive data are presented as the mean ± SD and median (IQR), where appropriate. Data were initially assessed for normality using the Shapiro–Wilk test. Most data exhibited a normal distribution, except for the SLR and PF MTU stiffness tests. One-way analysis of variance (ANOVA) or the Kruskal–Wallis test was used to compare baseline measurements and characteristics among the groups. An intention-to-treat approach was employed, with time and group effects analyzed using a mixed ANOVA [time (pre-, post-eight weeks of training, post-three-month follow-up) × group (five interventions and control)], encompassing the six interventions and each study arm of the three conditions. Additionally, two-way ANOVAs (time × intervention) were conducted separately for each arm of the three conditions. Post hoc multiple comparisons were conducted using the Bonferroni correction for significant interactions. The effect size (ES) was reported as the partial eta-squared for repeated measures [37], and as a Cohen *d* when the pairwise comparison was performed [38]. Statistical significance was set at an alpha level of 0.05. All statistical analyses were performed using the Statistical Package for the Social Sciences (SPSS) for Windows, version 24.0 (IBM Corp., Armonk, NY, USA).

## 3. Results

Compliance with training session attendance is illustrated in Figure 1. The baseline characteristics of the participants are detailed in Table 1. The RE and ECC groups had a lower proportion of men than the remaining groups (*p* < 0.001). Moreover, the participants in the SS group had a larger body mass than the RE and ECC groups (*p* = 0.013), and the participants in the SS and DS groups were taller than those in the ECC group (*p* = 0.04). In addition, the pre-intervention values for handgrip strength and SLR amplitude, the two indices used in selection for weakness and allocation into groups, exhibited substantial differences across the six groups, and resulted in different male/female (M/F) representation in each group.

### 3.1. Differences Between Groups at Baseline

The thickness of the BF muscle in the SS group was larger than that of the CON group (*p* = 0.034). Moreover, the resting and lengthened FLs of the BF muscle were greater in the RE group than in the CON group (*p* < 0.01). Furthermore, the FL of the MG in the SS group was greater than that in the CON group (*p* < 0.05). Participants with possible sarcopenia (i.e., those in the RE and ECC groups) exhibited a greater KF MTU compliance than those in the SS and DS groups (*p* < 0.05). In addition, there were differences among the groups regarding the normalized MIVC of the KE and KF muscles when adjusted for body mass, with the DS group exhibiting greater muscle strength than the RE group (*p* < 0.05). These differences were likely due to different representations of genders between groups.

### 3.2. Responses to Interventions

Body mass remained consistent across all groups throughout the study. Using an ITT approach with a mixed ANOVA, a substantial change in the results of the TUG test was observed over time [*F*(2,168) = 15.8, *p* < 0.001, *η*_p_^2^ = 0.16], without group effects and interaction. Specifically, the improvement in TUG performance was similar in all groups at the three-month follow-up, compared with the performance both pre-training and after eight weeks of training (0.34 and 0.23 s on average, respectively; *p* ≤ 0.001) (Table 2). In the analysis within each arm, an interaction effect for the stretching intervention [F(2,56) = 4.3, *p* = 0.018, *η*_p_^2^ = 0.13] was observed, indicating differences in change functional mobility between the SS and DS (*d* = 0.91, *p* = 0.003) groups after 8 weeks of training (Table 2), even though overall improvement was similar across groups after the 3-month follow-up.

Regarding changes in muscle strength, there was no significant interaction for the normalized MVC of the three muscle groups. However, there was a significant main effect of time for the KE [*F*(2,168) = 7.7, *p* = 0.001, *η*_p_^2^ = 0.08], KF [*F*(2,168) = 5.6, *p* = 0.005, *η*_p_^2^ = 0.06], and PF [*F*(2,168) = 8.3, *p* < 0.001, *η*_p_^2^ = 0.09] torque, as well as a group effect for the KE [*F*(5,84) = 2.8, *p* = 0.024, *η*_p_^2^ = 0.14] and KF [*F*(5,84) = 3.6, *p* = 0.005, *η*_p_^2^ = 0.18] torque. Moreover, there was an increase in peak torque for all three muscles after the eight-week training period in all groups, and these changes persisted in the PF muscle at the three-month follow-up (*p* = 0.009), as presented in Table 2. Within each study arm, similar changes in peak torque were observed between interventions—except for the comparison between the SS + ECC and CON groups, which revealed an interaction and group effect for KE (*d* = 0.81; *p* = 0.004 for the SS + ECC group), and group effects for KF and PF torque, as presented in Table 2.

In terms of muscle size, no significant interaction, group, or time effects were observed for the MT of the MG and BF muscles. However, the VL muscle exhibited both an interaction effect [*F*(10,168) = 2.2, *p* = 0.018, *η*_p_^2^ = 0.12] and a time effect [*F*(2,168) = 19.2, *p* < 0.001, *η*_p_^2^ = 0.19]. This was reflected by a substantial increase in the VL MT as a result of ECC training (*d* = 0.70, *p* = 0.018) (Table 3); however, no such change was noted in the remaining muscles investigated here, or with other interventions. This adaptation was diminished after the three-month follow-up, as the ECC group exhibited a reduction in the thickness of the VL muscle (*d* = 0.63, *p* < 0.05) compared with the measurements collected after eight weeks of training. A repeated measures ANOVA revealed no significant effects of interaction for MT within the study arms.

Last, the ultrasound measurements indicated a group effect for the three FL measurements, as observed in the baseline comparisons, independent of time effects. An interaction effect was found to be significant only for the BF FL measured at the end of the SLR test [*F*(10,168) = 2.4, *p* = 0.011, *η*_p_^2^ = 0.13], which was accompanied by a time effect [*F*(2,168) = 3.7, *p* = 0.027, *η*_p_^2^ = 0.042], with a marked increase in the FL in the DS intervention group (*d* = 0.77, *p* = 0.001), as detailed in Table 3. After the three-month follow-up, this change remained significant, with both the SS and DS groups exhibiting an increased FL compared with the pre-training measurements (*d* = 0.82, 0.90; all *p* < 0.01). When comparing each study arm, the results were similar across the six interventions, with changes only observed in individuals performed stretching exercises.

**Figure 2:**
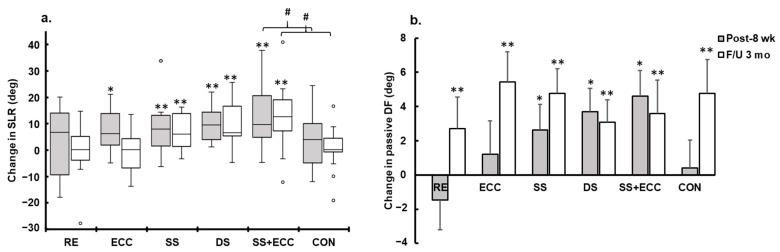
Absolute changes in hamstring (**a**) and calf (**b**) flexibility after eight weeks of home-based interventions and at three-month follow-up. Gray bar, post-eight weeks compared with baseline; blanked bar, three-month follow-up compared with pre-eight-week intervention. Data are presented as median differences and IQR for passive straight-leg raise (SLR), and as mean differences and SEM for passive ankle dorsiflexion (DF) (n = 15). * *p* < 0.05, ** *p* < 0.01. **^#^** Significant difference between interventions.

After eight weeks of training, a nonparametric analysis revealed that the SLR score was significantly improved in the ECC, SS, DS, and SS + ECC groups (all *p* < 0.05) (Figure 2a), although no group effect was noted. This improvement in the SLR test was still evident after the three-month follow-up in the SS, DS, and SS + ECC groups (all *p* < 0.05), with a less notorious improvement observed in the ECC group compared with the DS and SS + ECC groups (*p* < 0.05). Moreover, the SS + ECC group exhibited a significantly greater SLR angle at both the 8-week and 3-month follow-up, compared to the CON group (*p* < 0.05), in the study arm analysis.

No interaction or group effects were observed for calf flexibility measured during the PDF test. However, a significant time effect was found [*F*(2,168) = 21.5, *p* < 0.001, *η*_p_^2^ = 0.20]. The PDF angle increased in all groups following the eight-week intervention and at the three-month follow-up (Figure 2b). The analysis of each study arm revealed no interaction or group effects for calf flexibility measured during the PDF test. However, a significant time effect was observed in individuals with possible sarcopenia [*F*(2,56) = 9.3, *p* < 0.001, *η*_p_^2^ = 0.25], in those with hamstring tightness [*F*(2,56) = 10.2, *p* < 0.001, *η*_p_^2^ = 0.27], and in individuals with both weaknesses [*F*(2,56) = 3.4, *p* = 0.003, *η*_p_^2^ = 0.19].

**Figure 3:**
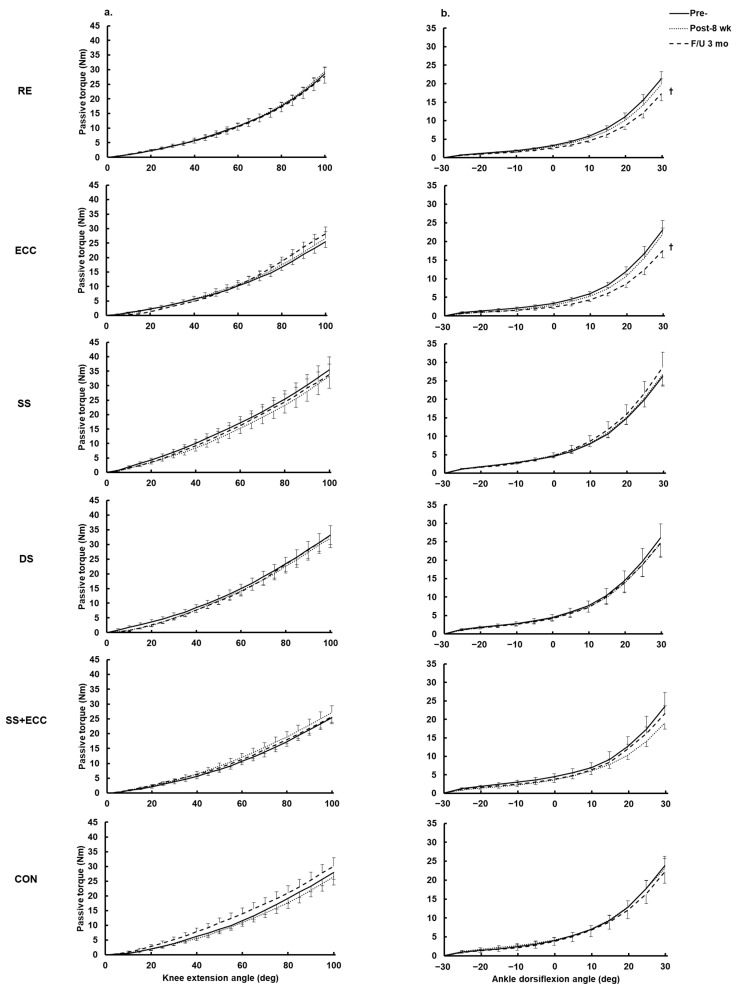
Passive torque–angle relationship of knee flexor (**a**) and ankle plantar flexor (**b**) MTU after eight weeks of home-based interventions and at three-month follow-up. Data are mean and SEM (n = 15). † Significant difference between pre-eight-week intervention and three-month follow-up.

A group effect was observed for the knee flexor MTU stiffness [*F*(5,84) = 21.5, *p* < 0.001, *η*_p_^2^ = 0.26], as shown in the baseline comparisons, independent of time or interaction effects (*p* > 0.05) (Figure 3a). There were no significant time, interaction, or group effects for knee flexor MTU stiffness across the three arms in this study (Figure 3a).

A nonparametric analysis revealed an absence of significant changes in PF MTU stiffness after the eight weeks of training across all study arms, with no differences observed between them. However, a decrease in PF MTU stiffness was later observed (at the three-month follow-up) in the RE group, from 0.69 to 0.57 N m/deg (*p* = 0.013), and in the ECC group, from 0.68 to 0.51 N m/deg (*p* = 0.035), independent of group difference (Figure 3b).

Finally, there were no significant differences among the groups in terms of satisfaction with the assigned exercise program, with average satisfaction scores of 2.6, 2.5, 2.4, 2.5, and 2.6 for the RE, ECC, SS, DS, and SS + ECC groups, respectively.

### 3.3. Physical Activity Volume and Continuous Exercise Sessions During Three-Month Follow-Up

After the three-month follow-up, the overall physical activity in the DS intervention group increased to 150 min per week (*p* = 0.049), whereas for the remaining intervention groups, it remained similar throughout the observation period. The number of continuous exercise sessions during the 3-month follow-up period after the eight weeks of intervention averaged 12, 13, 29, 18, and 20 sessions for the RE, ECC, SS, DS, and SS + ECC groups, respectively. Notably, the exercise frequency in the SS group was significantly higher than that in the RE group (*p* = 0.044).

## 4. Discussion

Aging is associated with the deterioration of various aspects of bodily function, and numerous studies have highlighted the benefits of muscle strengthening and stretching exercises in this context. In this study of older adult participants, special attention was given to addressing individual weaknesses. Home training programs were tailored to target elimination of deficiencies in strength, flexibility, or both. Unexpectedly, no significant differences were found between groups for mobility (TUG) and strength improvement, while it is noteworthy that improvements in mobility and leg strength did not occur simultaneously. The enhancement of leg flexibility was also random, with minor changes observed after the initial eight-week training period, followed by a more pronounced increase at the three-month follow-up. Overall, these improvements seem to be attributable to repeated measurement (learning effect) and/or the increased physical activity of participants, rather than being a direct effect of the home-based training.

The findings in present study partly contrast with previous research linking mobility improvements to strength training over 8–12 weeks [39,40]. Strength training did not provide a greater improvement in mobility upon re-testing compared to other groups. Moreover, the leg isometric strength improved in all groups over time, whereas muscle hypertrophy was observed exclusively in the RE and ECC groups after eight weeks of training, and disappeared within the follow-up period. These discrepancies might be explained by the relatively fit nature of our study population, as the participants were elderly, but otherwise healthy. Despite the inclusion of community-dwelling older adults with possible sarcopenia, as defined by the AWGS 2019 guidelines, the average TUG scores in all groups were above the normative values reported in a recent Thai study [41]. The TUG test is commonly used as a clinical assessment tool for functional performance, and it is widely utilized among older adults [42,43,44]. It appears that in fit elderly individuals, the TUG test is more sensitive to repeated measures than to strength gains. Therefore, the application of this program to individuals with lower performance levels requires further investigation.

Regarding flexibility changes resulting from the eight-week home-based programs applied in this study, the ECC, SS, DS, and SS + ECC interventions effectively improved hamstring flexibility, which is consistent with previous research [19,22,45,46]. Similar improvements in the PDF angle were observed in the SS, DS, and SS + ECC groups after the intervention. The flexibility improvement may be associated with increased physical mobility, as suggested by previous studies [20,21,22,47]. However, changes in flexibility and mobility did not always coincide, as no change in mobility was observed after static stretching, despite a significant increase in range of motion.

Unexpectedly, a further increase in the PDF angle was observed during the follow-up period. This could be attributed to continued physical activity or exercise (especially in the DS and SS groups), which contributed further to ROM gains. However, the improvement in leg flexibility in the two pure stretching groups did not differ significantly from that of the other groups during the follow-up period, and inconsistent changes in FL and no changes in MTU stiffness were observed. These results suggest that increased ROM is likely due to enhanced stretch tolerance induced by repeated measures, rather than to the home-based training itself. An increase in leg flexibility without corresponding changes in MTU stiffness highlights the importance of factors beyond passive MTU properties, such as muscle, deep fascia, or nerve tissue stiffness, in overall joint flexibility [17,48].

This study had several limitations. First, the unequal gender distribution across groups may have slightly influenced absolute values. Second, only one side of the body was assessed, leaving contralateral effects unexplored. Third, improvements in physical function may have involved muscle groups that were not specifically targeted, such as the gluteal muscles. A broader training approach may be necessary to fully assess the impact on overall physical function. Finally, baseline strength and flexibility performance influenced intervention allocation, making it difficult to separate the effects of initial fitness levels from those of the interventions. Nevertheless, we argue that this is not a critical issue, because one would generally expect the occurrence of an adaptation in response to any training program when dealing with training-naïve participants.

Clinical implications

The present study indicates that while most home-based exercise regimens improve functional performance, as shown in previous research [23,24], their benefits generally do not surpass those observed in non-exercised groups. However, strength-based programs demonstrate some superiority by inducing knee extensor muscle hypertrophy. Therefore, both elastic band or eccentric strength training may be recommended for older adults with suspected sarcopenia. Although delayed-onset muscle soreness (DOMS) is a common response to eccentric strength training, it typically occurs within the first few days or weeks of training, with subsequent bouts leading to reduced soreness over time [49].

## 5. Conclusions

Although increases in physical mobility, strength, and flexibility were observed after targeting muscle weakness or tightness with home-based programs, these improvements appeared to result from learning effects and increased physical activity, rather than the specific impact of the structured training. Strength-based programs, however, provided additional benefits, including short-lasting muscle hypertrophy in knee extensors. Therefore, both elastic band and eccentric strength training may be recommended for older adults with suspected sarcopenia.

## Figures and Tables

**Table 1:** Characteristics of participants.

	RE (N = 15)	ECC (N = 15)	SS (N = 15)	DS (N = 15)	SS + ECC (N = 15)	CON (N = 15)	*p*-Value
Gender (M/F)	1/14	1/14	9/6 ^r,e^	8/7 ^r,e^	6/9 ^r,e^	4/11 ^r,e^	<0.001
Age (years)	66.1 ± 4.7	67.5 ± 3.2	68.3 ± 4.3	65.9 ± 4.7	65.9 ± 3.9	66.9 ± 2.8	0.470
Body mass (kg)	55.1 ± 6.6 ^s^	52.8 ± 4.2 ^s^	65.2 ± 8.8	60.3 ± 10.2	55.8 ± 7.6	61.3 ± 14.3	0.004
Height (cm)	155.3 ± 6.0	153.9 ± 5.8	162.5 ± 9.2 ^e^	162.6 ± 7.4 ^e^	159.6 ± 7.4	159.1 ± 8.9	0.007
BMI (kg∙m^−2^)	22.9 ± 3.0	22.4 ± 1.8	24.7 ± 2.8	22.7 ± 2.6	21.9 ± 2.2	24.0 ± 3.9	0.062
PA volume (min∙wk^−1^) *	60 (150)	180 (240)	0 (210)	0 (90)	140 (200)	100 (375)	0.332
Handgrip strength (kg)	13 (6) ^s,d^	14 (4) ^s,d^	22 (12)	28 (11)	16 (10) ^s,d^	14 (6) ^s,d^	<0.001
SLR (deg)	92 (14)	85 (9)	75 (9) ^r, e^	71 (9) ^r,e^	68 (11) ^r,e^	72 (9) ^r,e^	<0.001

Data are mean ± SD, and median (interquartile range) for exercise volume, handgrip strength, and SLR. BMI, body mass index; PA, physical activity; RE, home-based resistance-band exercise; ECC, eccentric exercise; SS, static stretching exercise; DS, dynamic stretching exercise; CON, control; SLR, straight-leg raise. ^r^ significantly different from RE; ^e^ significantly different from ECC; ^s^ significantly different from SS; ^d^ significantly different from DS (all *p* < 0.05). * PA volume was recorded through self-reports, regardless of intensity of activity.

**Table 2:** Changes in Timed Up-and-Go (TUG) test performance and normalized maximum isometric voluntary contraction with interventions.

	RE (N = 15)	ECC (N = 15)	SS (N = 15)	DS (N = 15)	SS + ECC (N = 15)	CON (N = 15)
**TUG (s) T**ime effect (*p* < 0.001); **i**nteraction effect (*p* = 0.125); **g**roup effect (*p* = 0.154)						
**Pre-**	6.91 ± 1.03	6.65 ± 0.63	6.40 ± 1.11	6.45 ± 0.81	6.30 ± 0.80 ^G^	6.80 ± 0.86 ^G^
**Post-8 wk**	7.15 ± 0.91	6.51 ± 0.37	6.34 ± 1.23	5.82 ± 0.56 ^T^	6.00 ± 0.76	6.77 ± 1.04
**F/U 3 mo**	6.62 ± 1.20 ^T^	6.28 ± 0.63 ^T^	6.03 ± 1.14 ^T^	5.86 ± 0.70 ^T^	5.92 ± 0.66 ^T^	6.34 ± 0.65 ^T^
**Each arm**	T_effect_ (*p* = 0.007) I_effect_ (*p* = 0.176) G_effect_ (*p* = 0.159)		T_effect_ (*p* = 0.002) I_effect_ (*p* = 0.018) G_effect_ (*p* = 0.418)		T_effect_ (*p* = 0.010) I_effect_ (*p* = 0.645) G_effect_ (*p* = 0.029)	
**MIVC KE (Nm/BW) T**ime effect (*p* = 0.001); **i**nteraction effect (*p* = 0.185); **g**roup effect (*p* = 0.024)						
**Pre-**	1.53 ± 0.41	1.67 ± 0.37	1.80 ± 0.53	2.07 ± 0.39	1.87 ± 0.42 ^G^	1.80 ± 0.37 ^G^
**Post-8 wk**	1.68 ± 0.33 ^T^	1.85 ± 0.49 ^T^	2.04 ± 0.63	2.11 ± 0.45	2.14 ± 0.38 ^T^	1.74 ± 0.29
**F/U 3 mo**	1.74 ± 0.23	1.80 ± 0.51	1.87 ± 0.57	2.06 ± 0.36	2.09 ± 0.35	1.72 ± 0.29
**Each arm**	T_effect_ (*p* = 0.017) I_effect_ (*p* = 0.586) G_effect_ (*p* = 0.443)		T_effect_ (*p* = 0.057) I_effect_ (*p* = 0.264) G_effect_ (*p* = 0.307)		T_effect_ (*p* = 0.123) I_effect_ (*p* = 0.006) G_effect_ (*p* = 0.020)	
**MIVC KF (Nm/BW) T**ime effect (*p* = 0.005); **i**nteraction effect (*p* = 0.468); **g**roup effect (*p* = 0.005)						
**Pre-**	0.62 ± 0.17	0.70 ± 0.16	0.78 ± 0.25	0.88 ± 0.19	0.80 ± 0.15 ^G^	0.69 ± 0.17 ^G^
**Post-8 wk**	0.65 ± 0.11	0.76 ± 0.08	0.86 ± 0.31	0.89 ± 0.22	0.91 ± 0.13	0.70 ± 0.14
**F/U 3 mo**	0.71 ± 0.16 ^T^	0.77 ± 0.11 ^T^	0.87 ± 0.36	0.86 ± 0.22	0.88 ± 0.17	0.69 ± 0.18
**Each arm**	T_effect_ (*p* = 0.006) I_effect_ (*p* = 0.655) G_effect_ (*p* = 0.065)		T_effect_ (*p* = 0.368) I_effect_ (*p* = 0.244) G_effect_ (*p* = 0.668)		T_effect_ (*p* = 0.052) I_effect_ (*p* = 0.138) G_effect_ (*p* = 0.002)	
**MIVC PF (Nm/BW) T**ime effect (*p* = 0.001); **i**nteraction effect (*p* = 0.216); **g**roup effect (*p* = 0.111)						
**Pre-**	1.34 ± 0.29	1.38 ± 0.26	1.23 ± 0.33	1.39 ± 0.43	1.34 ± 0.31 ^G^	1.25 ± 0.32 ^G^
**Post-8 wk**	1.44 ± 0.37	1.39 ± 0.27	1.22 ± 0.32	1.59 ± 0.36 ^T^	1.52 ± 0.25	1.31 ± 0.21
**F/U 3 mo**	1.47 ± 0.39	1.44 ± 0.41	1.41 ± 0.44 ^T^	1.58 ± 0.35 ^T^	1.49 ± 0.30	1.29 ± 0.19
**Each arm**	T_effect_ (*p* = 0.261) I_effect_ (*p* = 0.669) G_effect_ (*p* = 0.842)		T_effect_ (*p* = 0.002) I_effect_ (*p* = 0.073) G_effect_ (*p* = 0.071)		T_effect_ (*p* = 0.136) I_effect_ (*p* = 0.618) G_effect_ (*p* = 0.017)	

Data are mean ± SD. RE, home-based resistance-band exercise; ECC, eccentric exercise; SS, static stretching exercise; DS, dynamic stretching exercise; SS + ECC, static stretching with eccentric exercise; CON, control; MIVC, maximal isometric voluntary contraction; KE, knee extensor; BW, body weight; KF, knee flexor; PF, plantar flexor. ^T^ Significant difference from pre-measurement for each study arm analysis. ^G^ Significant difference between exercise interventions for each study arm analysis. All *p* < 0.05.

**Table 3:** Changes in muscle thickness and fascicle length with six interventions.

	RE (N = 15)	ECC (N = 15)	SS (N = 15)	DS (N = 15)	SS + ECC (N = 15)	CON (N = 15)
**VL MT (cm) T**ime effect (*p* < 0.001); **i**nteraction effect (*p* = 0.018); **g**roup effect (*p* = 0.158)						
**Pre-**	1.86 ± 0.27	1.73 ± 0.27	1.98 ± 0.30	1.97 ± 0.39	1.75 ± 0.26	1.75 ± 0.39
**Post-8 wk**	1.94 ± 0.35 ^T^	1.88 ± 0.11 ^T^	2.05 ± 0.32	1.96 ± 0.34	1.83 ± 0.34	1.80 ± 0.29
**F/U 3 mo**	1.82 ± 0.38	1.59 ± 0.16	1.96 ± 0.27	1.96 ± 0.42	1.77 ± 0.32	1.83 ± 0.25
**Each arm**	T_effect_ (*p* = 0.001) I_effect_ (*p* = 0.105) G_effect_ (*p* = 0.122)		T_effect_ (*p* = 0.507) I_effect_ (*p* = 0.476) G_effect_ (*p* = 0.079)		T_effect_ (*p* = 0.366) I_effect_ (*p* = 0.729) G_effect_ (*p* = 0.920)	
**BF MT (cm) T**ime effect (*p* = 0.232); **i**nteraction effect (*p* = 0.521); **g**roup effect (*p* = 0.003)						
**Pre-**	1.61 ± 0.30	1.60 ± 0.30	1.74 ± 0.23	1.69 ± 0.32	1.67 ± 0.24 ^G^	1.42 ± 0.26 ^s,G^
**Post-8 wk**	1.60 ± 0.29	1.53 ± 0.25	1.79 ± 0.35	1.66 ± 0.26	1.63 ± 0.17	1.35 ± 0.23
**F/U 3 mo**	1.57 ± 0.22	1.48 ± 0.18	1.77 ± 0.22	1.68 ± 0.18	1.62 ± 0.17	1.45 ± 0.25
**Each arm**	T_effect_ (*p* = 0.155) I_effect_ (*p* = 0.558) G_effect_ (*p* = 0.480)		T_effect_ (*p* = 0.892) I_effect_ (*p* = 0.688) G_effect_ (*p* = 0.340)		T_effect_ (*p* = 0.455) I_effect_ (*p* = 0.538) G_effect_ (*p* = 0.001)	
**MG MT (cm) T**ime effect (*p* = 0.980); **i**nteraction effect (*p* = 0.232); **g**roup effect (*p* = 0.248)						
**Pre-**	1.56 ± 0.22	1.47 ± 0.17	1.60 ± 0.30	1.60 ± 0.25	1.49 ± 0.25	1.44 ± 0.19
**Post-8 wk**	1.56 ± 0.25	1.50 ± 0.13	1.61 ± 0.27	1.56 ± 0.27	1.45 ± 0.26	1.54 ± 0.17
**F/U 3 mo**	1.56 ± 0.21	1.41 ± 0.15	1.64 ± 0.27	1.59 ± 0.27	1.47 ± 0.24	1.50 ± 0.20
**Each arm**	T_effect_ (*p* = 0.159) I_effect_ (*p* = 0.155) G_effect_ (*p* = 0.144)		T_effect_ (*p* = 0.743) I_effect_ (*p* = 0.682) G_effect_ (*p* = 0.733)		T_effect_ (*p* = 0.618) I_effect_ (*p* = 0.122) G_effect_ (*p* = 0.733)	
**BF FL at rest (cm) T**ime effect (*p* = 0.274); **i**nteraction effect (*p* = 0.450); **g**roup effect (*p* = 0.006)						
**Pre-**	10.77 ± 2.66	8.99 ± 2.25	8.74 ± 2.32	8.71 ± 1.95	9.63 ± 2.70 ^G^	7.85 ± 1.98 ^r,G^
**Post-8 wk**	10.40 ± 1.86	9.63 ± 2.49	9.54 ± 2.33	9.16 ± 1.66	9.50 ± 1.97	7.53 ± 1.76
**F/U 3 mo**	10.80 ± 2.20	9.27 ± 1.97	9.30 ± 2.13	9.76 ± 2.11	9.16 ± 1.63	7.62 ± 2.32
**Each arm**	T_effect_ (*p* = 0.904) I_effect_ (*p* = 0.422) G_effect_ (*p* = 0.056)		T_effect_ (*p* = 0.049) I_effect_ (*p* = 0.457) G_effect_ (*p* = 0.988)		T_effect_ (*p* = 0.626) I_effect_ (*p* = 0.833) G_effect_ (*p* = 0.010)	
**BF FL at the end of SLR (cm) T**ime effect (*p* = 0.010); **i**nteraction effect (*p* = 0.036); **g**roup effect (*p* < 0.001)						
**Pre-**	16.68 ± 2.35	14.33 ± 3.09	13.86 ± 2.34	12.28 ± 3.01	14.33 ± 2.98 ^G^	11.47 ± 2.15 ^G^
**Post-8 wk**	15.54 ± 2.10	14.49 ± 2.70	14.13 ± 2.54	13.90 ± 3.25 ^T^	15.32 ± 1.02	11.27 ± 2.00
**F/U 3 mo**	16.08 ± 2.54	14.41 ± 3.31	15.30 ± 2.33 ^T^	14.54 ± 3.68 ^T^	15.48 ± 1.72	11.02 ± 3.01
**Each arm**	T_effect_ (*p* = 0.486) I_effect_ (*p* = 0.269) G_effect_ (*p* = 0.080)		T_effect_ (*p* = 0.001) I_effect_ (*p* = 0.250) G_effect_ (*p* = 0.397)		T_effect_ (*p* = 0.699) I_effect_ (*p* = 0.154) G_effect_ (*p* = 0.001)	

Data are mean ± SD. RE, home-based resistance-band exercise; ECC, eccentric exercise; SS, static stretching exercise; DS, dynamic stretching exercise; CON, control; VL, vastus lateralis; MT, muscle thickness; BF, biceps femoris; MG, medial gastrocnemius; FL, fascicle length; SLR, straight-leg raise. ^T^ Significant difference from pre-measurement for each study arm analysis. ^G^ Significant difference between exercise interventions for each study arm analysis. ^s^ significantly different from SS. ^r^ significantly different from RE. All *p* < 0.05.

## Data Availability

Data are available upon request.

## Abbreviations

The following abbreviations are used in this manuscript:

BF	Biceps femoris
DS	Dynamic stretching
ECC	Eccentric exercise
EI	Echo intensity
FL	Fascicle length
MIVC	Maximum isometric voluntary contraction
MG	Medial gastrocnemius
MT	Muscle thickness
MTU	Musculotendinous unit
PDF	Passive ankle dorsiflexion
RE	Home-based resistance-band exercise
ROM	Range of motion
RPE	Rate of Perceived Exertion
SLR	Straight-leg raise
SS	Static stretching
TUG	Timed Up-and-Go
VL	Vastus lateralis
